# The analysis of ecological security and tourist satisfaction of ice-and-snow tourism under deep learning and the Internet of Things

**DOI:** 10.1038/s41598-024-61598-y

**Published:** 2024-05-10

**Authors:** Baiju Zhang

**Affiliations:** The Tourism College of Changchun University, Jilin Northeast Asia Research Center On Leisure Economics, Jilin Province Research Center for Cultural Tourism Education and Enterprise Development, Changchun Industry Convergence Research Center of Culture and Tourism, Changchun Ice and Snow Industry Research Institute, Changchun, 130607 China

**Keywords:** Deep learning, Internet of Things technology, Ice-and-snow tourism, Ecological security, Tourist satisfaction, Computational science, Computer science

## Abstract

This paper aims to propose a prediction method based on Deep Learning (DL) and Internet of Things (IoT) technology, focusing on the ecological security and tourist satisfaction of Ice-and-Snow Tourism (IST) to solve practical problems in this field. Accurate predictions of ecological security and tourist satisfaction in IST have been achieved by collecting and analyzing environment and tourist behavior data and combining with DL models, such as convolutional and recurrent neural networks. The experimental results show that the proposed method has significant advantages in performance indicators, such as accuracy, F1 score, Mean Squared Error (MSE), and correlation coefficient. Compared to other similar methods, the method proposed improves accuracy by 3.2%, F1 score by 0.03, MSE by 0.006, and correlation coefficient by 0.06. These results emphasize the important role of combining DL with IoT technology in predicting ecological security and tourist satisfaction in IST.

## Introduction

### Research background and motivations

Ice-and-Snow Tourism (IST), as a special and attractive form of tourism, has gradually emerged globally in recent years. The magnificent scenery, diverse ice and snow activities, and unique cultural experiences in the snowy region have attracted more and more tourists, bringing important opportunities for local economic development and tourism prosperity^1–3^. However, with the rapid development of IST, a series of potential challenges and problems are also becoming increasingly apparent^4,5^. Firstly, the ecological security issues of IST are increasingly attracting attention^6–8^. As the number of tourists increases, the impact of tourism activities on the natural environment gradually increases^9^. For example, tourist behavior may disrupt the local ecological balance, damage the habitats of animals and plants, and even lead to soil erosion and water pollution^10^. Additionally, external factors, such as climate change, also have an impact on the ice and snow environment, exacerbating the vulnerability of ecosystems^11–13^. Therefore, ecological security and tourists' satisfaction have become important issues worthy of attention. Ecological security refers to protecting and maintaining the safety and stability of the natural environment in tourism activities to ensure the health of the ecosystem and the maintenance of biodiversity. This involves the protection of animal and plant habitats, the maintenance of water and air quality, the prevention of pollution of land and water resources, and the reduction of destructive impact on natural ecosystems. Tourists' satisfaction refers to tourists' feelings and evaluation of tourist destinations, services and experiences during their travels. This reflects tourists' satisfaction with tourism activities, including the attraction of tourist destinations, service quality, tour experience, facilities and equipment, cultural experience, and convenient transportation. Therefore, how to achieve sustainable development of IST while ensuring the ecological security of ice and snow has become an urgent problem to be solved^14–16^.

Secondly, the improvement of tourist satisfaction is also an important direction for the development of IST industry. With the rapid development of information technology, tourists' expectations and demands for tourist destinations are also constantly increasing^17^. Tourists pay more attention to personalized travel experiences, hoping to receive higher quality services, more diverse activity choices, and more convenient ways to obtain information during the travel process^18–20^. Therefore, how to effectively meet the diverse needs of tourists and enhance their tourism experience has become an important challenge faced by IST enterprises and destination managers^21–23^. This paper combines Internet of Things (IoT) sensor data with Deep Learning (DL) models to achieve real-time monitoring and risk prediction of IST environments, providing scientific basis for tourism managers.

### Research objectives

This paper aims to explore how to improve the ecological security level of IST and tourist satisfaction from the perspective of DL, combined with IoT technology. The specific research objectives include: 1) Building a comprehensive research method that integrates IoT sensor data with DL models to achieve real-time monitoring and risk prediction of IST environments. 2) Designing and implementing DL models to improve the accuracy of predicting changes in ice and snow environments and extreme weather events and provide support for ecological security decision-making. 3) Evaluating the ecological security index of IST using the predicted results of the model to explore the correlation between the two combined with tourist satisfaction survey data.

This paper is unique and innovative in the following aspects:

1) Establishment of comprehensive research method: This paper is committed to establishing a comprehensive research method, which combines the sensor data of the Internet of Things (IoT) with the deep learning (DL) model. This comprehensive method enables people to realize real-time monitoring and risk prediction of ice and snow tourism environment. By making full use of IoT technology, people can collect a large amount of environmental data and analyze it with DL model to more accurately evaluate environmental changes and potential ecological security risks.

2) Design and implementation of DL model: DL model is adopted to improve the prediction accuracy of ice and snow environmental changes and extreme weather events. By using DL technology, people can better capture the pattern of complex environmental changes and provide reliable support for ecological security decision-making. This method is unique in improving the prediction of environmental change and providing a more reliable information base for decision makers.

3) Correlation analysis between the evaluation of ecological security index and tourists' satisfaction: It not only pays attention to the technical level of ecological security, but also evaluates the ecological security index of IST by combining the results predicted by the model with the survey data of tourists' satisfaction. This correlation analysis is helpful for a more comprehensive understanding of the relationship between ecological security level and tourist experience, and provides strong support for formulating comprehensive IST management strategies. Through these research objectives and methods, this paper not only emphasizes technological innovation, but also pays more attention to achieving positive ecological security and improving tourists' satisfaction in practical application. It is believed that this comprehensive method and unique perspective will bring beneficial enlightenment and promotion to the field of IST management and ecological protection.

## Literature review

In recent years, with the rapid development of information technology and artificial intelligence, research in IST has also received increasing attention^24^. Satrya et al.^25^ discussed the attitude, behavior and experience of millennials towards ecotourism. The results showed that millennials paid more attention to sustainability, cultural experience and natural environment in ecotourism, which provided an important reference for the development of IST. Hong-Min et al.^26^ discussed the IST and its sustainable development in China. The study analyzed the impact of IST on China's economy and environment from a new perspective of poverty alleviation. The results showed that IST provided important economic opportunities for China, and also promoted the development and environmental protection of poverty-stricken areas. Cai et al.^27^ analyzed the spatial relationship and coordinated development between IST and other related industries in Jilin Province. The results showed that there was a close spatial correlation between the development of IST in Jilin Province and other industries, which provided an important reference for the integration of IST industrial chain. Sun^28^ used the symmetry analysis method to explore the influence of tourism consumers' cognitive and emotional attitudes on the choice and experience of tourism destinations. The results revealed tourists' preferences and behavior patterns in the decision-making process, which provided theoretical support for IST marketing and service.

Liu and Guo^29^ analyzed the image perception of IST destinations in China. They further studied the impact of the Winter Olympics on destination image through online text analysis of data from multiple online tourism platforms, such as Ctrip, Qunar, and Meituan. Zhao et al.^30^ conducted research and analysis on the evaluation of the competitiveness of IST. They used an improved fuzzy neural network algorithm to process the system flowchart of IST development through the functions and characteristics of the IST power system. Huang et al.^31^ used social learning theory to identify the determining factors of community ecotourism in Taiwan using international standards. This basic theory of social psychology comprehensively analyzed three analytical perspectives (ecotourism, destinations, and accommodation) and four basic issues of the Global Sustainable Tourism Council. The new geospatial decision support system developed by Mileti et al.^32^ on the geospatial network infrastructure had substantive interdisciplinary core functions and could provide valuable web-based business tools.

Although significant achievements have been made in the application of DL and IoT technology in the tourism field, research in IST is still relatively scarce. At present, research on how to effectively combine DL and IoT technology to improve the ecological security and tourist satisfaction of IST is still relatively limited. Especially in practical scenarios, more exploration and experimentation are needed on how to build integrated systems, how to handle large-scale environmental data, and how to solve problems, such as network transmission and data privacy. This paper provides innovative solutions in real-time monitoring of ice and snow environments, improving ecological security decisions, and optimizing tourist experiences. Meanwhile, it strives to combine ecological security with tourist satisfaction, explore the correlation between the two, provide scientific basis for tourism managers, and fill the research gap in this area.

## Research methodology

### Overall methodological framework and data collection

**Figure 1:**
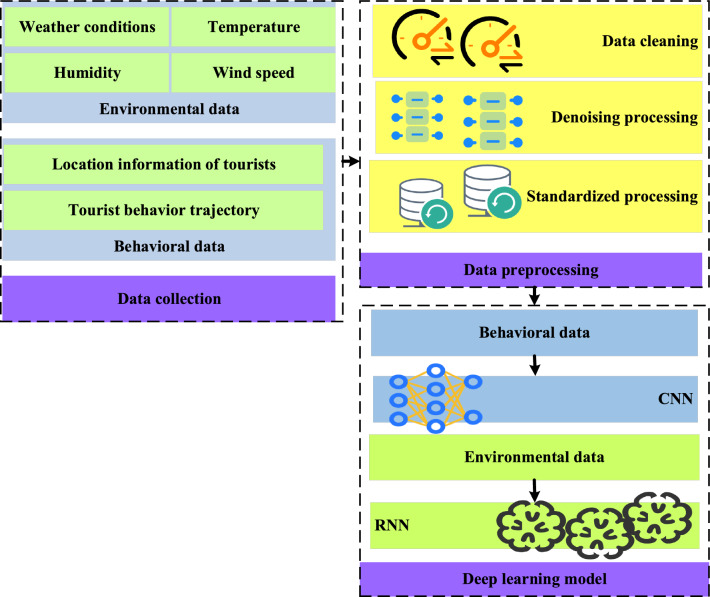
Composition of the research method framework.

The overall framework of the method is shown in Fig. 1. In this framework, DL and IoT technology are combined to achieve comprehensive research on the ecological security and tourist satisfaction of IST.

From Fig. 1, this framework covers key steps, such as data collection, application of DL models, and integration of IoT data. Firstly, data collection is the foundation of research. Information is obtained from multiple data sources to gain a comprehensive understanding of the IST environment. Specifically, IoT sensors, including weather stations and soil moisture sensors, are deployed to obtain real-time environmental information, such as weather conditions, temperature, humidity, and wind speed. Besides, the location information, behavior trajectory, and visitor satisfaction feedback of tourists are also collected through mobile devices, Global Positioning System (GPS) tracking, and other methods. These data provide basic information for research.

Data preprocessing is a key step in ensuring data quality. The collected raw data is cleaned, denoised, and standardized to ensure data consistency and accuracy. In the data cleaning step, missing values, abnormal values and duplicate values in the dataset are identified and processed, including filling missing values or deleting samples containing missing values to process missing values, standardizing or eliminating abnormal values beyond a reasonable range to process abnormal values, and identifying and deleting duplicate records to process duplicate values. In the data denoising step, smoothing technology (such as moving average or median filtering) is used to smooth the noise in time series data, and clustering or outlier detection algorithm is used to identify and eliminate outliers. In the data standardization step, the numerical features are scaled to have the same range or unit. The category features are coded or digitized to facilitate model processing. Time series data are changed by difference or percentage to eliminate the trend and seasonal influence. For example, outlier detection and missing value processing are performed on environmental data, and trajectory smoothing and denoising are performed on tourist behavior data. This provides high-quality data support for the application of DL models.

Finally, a DL model is used to analyze the data to achieve a comprehensive study on the ecological security and tourist satisfaction of IST. Specifically, the Convolutional Neural Network (CNN) is used to process environmental data and extract environmental features, and the Recurrent Neural Network (RNN) is used to analyze tourist behavior data and extract time series features. The two networks fuse the extracted features to achieve comprehensive analysis. In summary, this method framework combines IoT technology with DL methods to address the issues of ecological security and tourist satisfaction in IST. Through data collection, preprocessing, and the application of DL models, the IST environment can be comprehensively understood to provide scientific basis for tourism managers.

### DL model architecture and integration with IOT Technology

Compared with other methods such as traditional machine learning algorithm and statistical analysis, the advantages of deep learning model in revealing the correlation between environmental data and tourist behavior data are that it can deal with complex nonlinear relationships, automatically learn features, adapt to large-scale data, and support end-to-end learning. This enables the deep learning model to capture the complex correlation between environmental data and tourist behavior data more accurately, and provides more reliable analysis tools and prediction capabilities for the study of ecological security and tourist satisfaction of IST.

**Figure 2:**
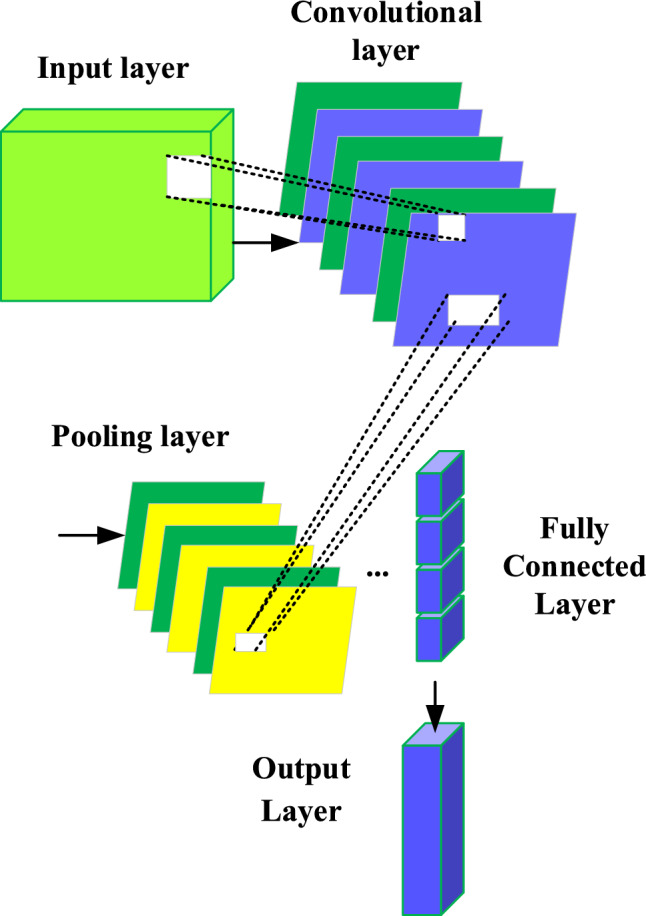
Network structure of CNN.

Therefore, this paper uses DL model to analyze environmental data and tourist behavior data, reveals the correlation between them, and realizes a comprehensive study of IST ecological security and tourist satisfaction. CNN is used to process environmental data to extract environmental features. CNN performs well in image processing to automatically learn and capture spatial features in data. The architecture of CNN includes convolutional layer, pooling layer, and fully connected layer (as shown in Fig. 2).

In the convolutional layer, the convolutional kernel slides over the data to extract features at different scales. Each convolutional kernel detects different local patterns to capture spatial features in the data. The pooling layer is used to reduce data dimensions and preserve key features. The fully connected layer maps the extracted features to the final output. These outputs represent key features in environmental data.

RNN performs well in processing sequential data and can effectively model temporal dependencies to capture time series information in tourist behavior data. Specifically, the characteristic of RNN is that each time step has a hidden state used to store information from previous time steps. This allows RNN to naturally process sequence data and capture temporal patterns in the data.

The different architectures of CNN-RNN have a significant impact on the performance and effect of the model. In the serial structure, CNN and RNN are connected in sequence, and deal with space and time information respectively, but there may be delay and loss in information transmission. In the parallel structure, CNN and RNN process the input data independently, and then fuse the feature representation, which makes the information exchange more effective, but it requires more computing resources and training time. The hybrid structure is a compromise, which can not only deal with different aspects of information, but also balance the calculation efficiency and information transmission efficiency. Therefore, in this paper, the collected data includes environmental data and tourist behavior data, CNN and RNN are used to extract the corresponding features respectively, so CNN-RNN parallel architecture is adopted to build the model.

**Figure 3:**
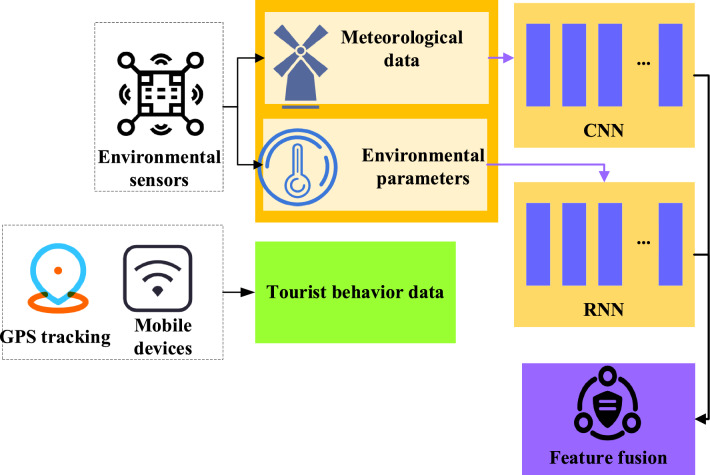
Schematic diagram of the fusion framework of the IoT and DL.

Finally, IoT data is integrated with DL models. IoT sensor data serves as input for DL models. Environmental data is processed using CNN to extract environmental features, such as temperature, humidity, and wind speed. CNN can capture the correlation between different environmental features and provide important clues for analysis. The behavior data of tourists is processed by RNN to reveal the evolution trend of tourists' behavior, such as the number of tourists and activity trajectory. RNN can capture the time series patterns of tourist behavior, providing a temporal dimension of understanding for research. Through this integration, environment and tourist behavior factors can be simultaneously considered to reveal their impact on the ecological security and tourist satisfaction of IST. Ultimately, the features extracted by the two networks are fused to form a comprehensive analysis result. The fusion labels are IST ecological security and tourist satisfaction to comprehensively evaluate and analyze the impact of environmental data and tourist behavior data on IST. Figure 3 shows the architecture of the DL model and the integration with IoT technology.

Meanwhile, in this study, the classification criteria of IST ecological security and tourist satisfaction are shown in Table 1.

**Table 1:** IST ecological security and tourist satisfaction classification standard table.

Index	Very satisfied	Satisfied	General	Unsatisfied	Very unsatisfied
Temperature	> 10 °C	5–10 °C	0–5 °C	− 5 to 0 °C	< − 5 °C
Humidity	30–40%	40–50%	50–60%	60–70%	> 70%
Snowfall	Light snow	Moderate snow	Heavy snow	Snowstorm	Hail
Tourist’s stay time	> 4 h	2–4 h	1–2 h	30 min–1 h	< 30 min

### Model training, validation, and performance evaluation

**Figure 4:**
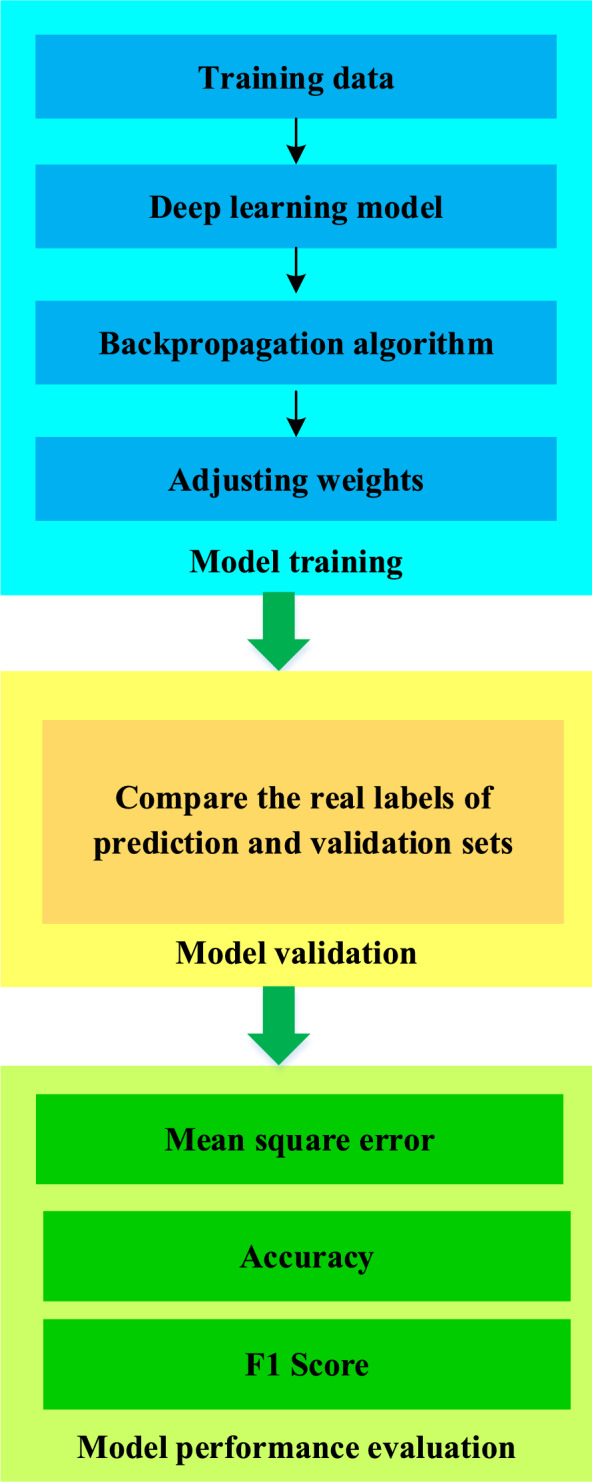
Model training, validation, and performance evaluation process.

The training of the model adjusts the weight of the model through a backpropagation algorithm to gradually adapt to the data^33–35^. Here, labeled data is used for supervised learning to optimize the model by minimizing the loss function^36^. The loss function compares the model's predictions with the actual labels, reflecting the model's prediction error^37–39^. In the training process, the parameters of the model are updated by the back propagation algorithm, so that it can better fit the data and accurately predict the IST ecological security and tourist satisfaction. In the training process, the loss function is usually used to measure the difference between the predicted results of the model and the real labels, and then the loss function is minimized by the random gradient descent algorithm. To evaluate the performance of the model on unprecedented data, model validation is required. The validation set is data that is partitioned from training data but has not been used during the training process^40–43^. During the validation process, the predictions of the model are compared with the true labels of the validation set. It is possible to determine whether the model is overfitting or under-fitting and whether adjustments are needed by observing the performance on the validation set. The schematic diagram of the specific model training, validation, and performance evaluation process is displayed in Fig. 4.

Figure 4 shows the changes in the loss function during model training and how to use validation sets to evaluate the performance of the model. Various performance indicators are used to measure the predictive ability of the model in terms of ecological security and tourist satisfaction in IST. The optimal model configuration can be found to achieve optimal performance by trying different combinations of hyper-parameters. This process requires cross validation to ensure that the model performs consistently across different subsets of data. Finally, the model configuration with the best performance is selected and used for subsequent experiments and analysis. Through the process of model training, verification, and performance evaluation, the effect of the DL model in analyzing the ecological security and tourist satisfaction of IST is determined to provide scientific support for subsequent research and decision-making.

### Ethics approval

The studies involving human participants were reviewed and approved by The Tourism College of Changchun University, Jilin Northeast Asia Research Center on Leisure Economics Ethics Committee (Approval Number: 2022.49584856). The participants provided their written informed consent to participate in this study. All methods were performed in accordance with relevant guidelines and regulations.

## Experimental design and performance evaluation

### Datasets collection

In order to ensure the smooth data collection, we have cooperated closely with different organizations and individuals to obtain diverse and real data and ensure that the research is more convincing. The close cooperation with local weather stations have been established. These weather stations provide abundant meteorological data, including temperature, humidity and snowfall. Meanwhile, cooperative relations with many mobile device data providers have been established. The applications and cooperation agreements adopted by these partners provide the data of mobile devices used by tourists during IST^44–46^. This includes information such as GPS trajectory and activity range, which helps to capture the activity patterns of tourists at different times and places. At the same time, it works closely with the professional sensor arrangement organization of the IoT to deploy sensors in the key areas of IST. These institutions are responsible for ensuring the correct installation and normal operation of sensors to obtain accurate and comprehensive environmental data. Through this arrangement, people can monitor the ecological environment of IST more comprehensively and provide more detailed data support for our research.

These data from IoT sensors are introduced to obtain more detailed and comprehensive environmental information. These sensors are distributed in key areas of IST, including ecologically sensitive areas and tourist activity hotspots. The types of sensors include environmental sensors (measuring air quality, soil moisture, etc.), image sensors (capturing environmental scenes), sound sensors, etc. Such diverse sensor networks are helpful to monitor the ecological environment of IST more comprehensively. The acquisition frequency of sensor data varies according to the sensor type. For example, an environmental sensor measures air quality once every hour, while an image sensor captures an environmental scene once every minute.

In order to ensure the comprehensive effectiveness of the model in predicting the ecological security of IST environment and tourists' satisfaction, data are obtained from multiple data sources, covering the IST season in the past two years. The main data sources and their characteristics are shown in Table 2.

**Table 2:** Data source and its characteristic tag table.

Data content	Description	Sign	Data acquisition method	Acquisition frequency
Temperature	Real-time temperature data in IST area	Environmental data	Deploy weather station sensors	Every five minutes
Humidity	Real-time humidity data in IST area	Environmental data	Deploy weather station sensors	Every five minutes
Snowfall	Real-time snowfall data in IST area	Environmental data	Deploy weather station sensors	Every five minutes
GPS trajectory	The GPS data of tourists' moving trajectory, including position coordinates and time stamps	Tourist behavior data	Use mobile applications or GPS tracking devices to collect	Every five minutes
Scope of activities	Regional data of tourists' activities during IST	Tourist behavior data	Use mobile applications or GPS tracking devices to collect	Every five minutes
Air quality	Real-time air quality data measured by environmental sensors in IST area	Environmental data	Deploy environmental sensors	Every five minutes
Soil moisture	Real-time soil moisture data measured by environmental sensors in IST area	Environmental data	Deploy soil moisture sensors	Every five minutes
Environmental scene	Environmental scene data captured by image sensor in IST area	Environmental data	Deploy image sensors	Every five minutes
Tourist behavior path	Travel path data of tourists during IST, including time stamp and location coordinates	Tourist behavior data	Use mobile applications or GPS tracking devices to collect	Every five minutes
Dwell time	Time data of tourists staying in different locations of IST	Tourist behavior data	Use mobile applications or GPS tracking devices to collect	Every five minutes
Activity	Data of activities that tourists participated in during IST, such as skiing and snowmobiling	Tourist behavior data	Use mobile applications or GPS tracking devices to collect	Every five minutes

In Table 2, in order to collect data, firstly, environmental sensors, including weather stations and soil moisture sensors, are deployed in the key areas of IST. These sensors collect data every five minutes, including environmental information such as temperature, humidity and snowfall. Meanwhile, a cooperative relationship with partners is established to obtain data on mobile devices used by tourists. The GPS trajectory and activity range of tourists are collected through mobile applications or GPS tracking devices, and these data are also collected every five minutes. The collected environmental data and mobile device data are transmitted to the data center for storage and processing. In the data center, a database is established to uniformly store environmental data and mobile device data, and the data is cleaned, denoised and standardized to ensure the quality and consistency of the data. Next, the collected data is processed based on the data with the lowest frequency to ensure the synchronization of the data. In this data collection, because it involves the data collection of tourists' behavior, it is strictly protected for privacy and security. Firstly, before collecting the data of mobile devices, the informed consent of users must be obtained. The purpose, usage and protection measures of data collection are clearly explained to users, and their clear consent is obtained. Secondly, a strict data authority and access control mechanism is established to restrict only authorized personnel to access and process mobile device data to ensure that only people with the necessary permissions can view and use the data. Third, when collecting mobile device data, anonymous and desensitized personal identity and sensitive information are processed to protect users' privacy. Finally, the data of mobile devices are encrypted to ensure the security of data during transmission and storage, and only authorized personnel can decrypt and access the data. Through these data, people can better understand the behavior of tourists under different environmental conditions to more accurately evaluate ecological security and tourist satisfaction.

In order to train and evaluate the model, the whole data set is divided into training set, verification set and test set in time sequence, and the ratio is 14:3:3. The purpose of this division is to maintain the balance of data sets and ensure that the model can obtain sufficient information in training and evaluation. Although the continuity of time sequence is emphasized, it will also introduce a certain degree of randomness to ensure the adaptability of the model to various situations. For example, by randomly selecting a certain proportion of data samples to increase the diversity of data, the complexity of IST environment can be better captured. At the same time, when dividing data sets, the problem of data leakage is avoided. This includes not including future information in the verification and test set in the training set.

### Experimental environment

A high-performance computer is used in the experiment, and a graphics processor suitable for DL tasks is also configured to ensure efficient training and evaluation of DL models. Table 3 lists the detailed settings of the experimental environment.

**Table 3:** Experimental environment configuration.

Hardware	Device	Specification
	Computer model	XYZ SuperCompute 5000
	Processor	Intel Core i9-10900 K @ 3.7 GHz
	Memory	32 GB DDR4 RAM
	Graphics processing unit	NVIDIA GeForce RTX 3090
	Storage	1 TB SSD + 2 TB HDD
Software environment	Software	Version
	Operating system	Ubuntu 20.04
	Python	3.8
	TensorFlow	2.5.0
	PyTorch	1.9.0
	CUDA	11.1
	cuDNN	8.2.1

### Parameters setting

During the model training process, a series of parameter adjustments are made to find the optimal model configuration. Table 4 gives some of the parameters set in the experiment.

**Table 4:** Parameter settings.

Parameter	Value range	Final setting value
Learning rate	0.001–0.1	0.01
Batch size	16, 32, and 64	32
Number of layers	1–5	3

### Performance evaluation

To quantify the performance of the model, a series of evaluation indicators are used. In studying ecological security and tourist satisfaction in IST, the following indicators are used to evaluate the performance of the model.

1) Mean Squared Error (MSE). It measures the average error between the predicted and actual values of the model and is suitable for regression problems^47^.

1 $$MSE=\frac{1}{N}\sum_{i=1}^{N}{({y}_{i}-\widehat{{y}_{i}})}^{2}$$

In Eq. (1), *N* is the number of samples, ${y}_{i}$ is the actual value, and $\widehat{{y}_{i}}$ is the predicted value of the model.

2) Accuracy. It is used for classification problems to represent the correct sample proportion predicted by the model.

2 $$Accuracy=\frac{\text{Number of Correct Predictions}}{\text{Total Number of Predictions}}\times 100\%$$

3) F1 score. It considers both precision and recall and is suitable for the problem of imbalanced category distribution.

3 $$F1=2\times \frac{{\text{Precision}}\times {\text{Recall}}}{{\text{Precision}}+{\text{Recall}}}$$

4 $${\text{Precision}}=\frac{\text{True Positives}}{\text{True Positives+False Positives}}$$

5 $$Recall=\frac{\text{True Positives}}{\text{True Positives+False }{\text{Ne}}{\text{gatives}}}$$

4) Correlation coefficient. It measures the linear relationship between variables and is used to understand the correlation between variables.

6 $$ r = \frac{{\sum \left( {x_{i} - \overline{x}} \right)\left( {y_{i} - \overline{y}} \right)}}{{\sqrt {\sum (x_{i} - \overline{x})^{2} \sum (y_{i} - \overline{y})^{2} } }} $$

In Eq. (6), ${x}_{i}$ and ${y}_{i}$ are the value of the variable, and $\overline{x}$ and $\overline{y}$ are the mean of the variable.

**Figure 5:**
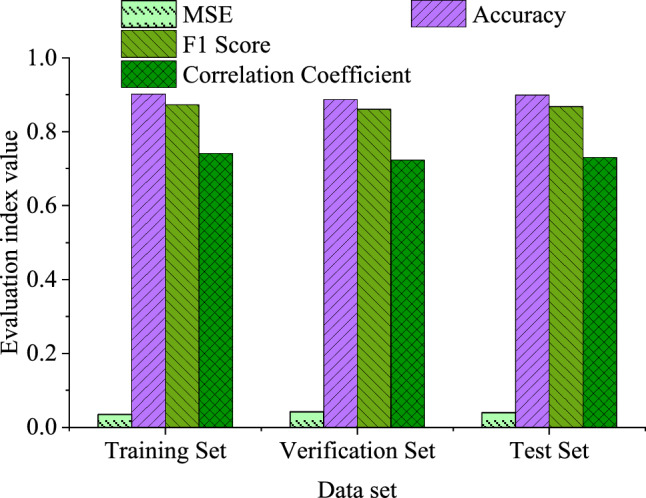
Indicator results of different datasets in the experiment.

The specific results of various performance indicators in the experiment are plotted in Fig. 5.

**Figure 6:**
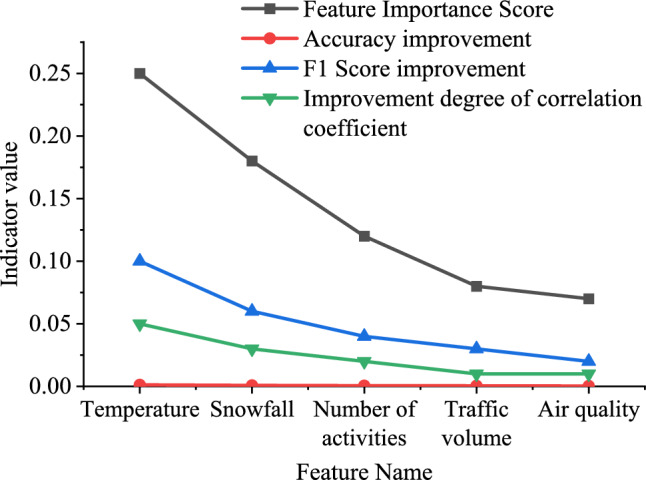
The relationship between different characteristics of IST.

As shown in Fig. 5, the MSE on the training set is 0.035, while the MSEs on the validation and test sets are 0.042 and 0.040, respectively. This indicates that the model achieves lower prediction errors on the training set and maintains good generalization performance on the validation and test sets. The accuracy of the model on the training set, validation set, and test set is 90.2%, 88.7%, and 89.9%, respectively. The model can achieve high accuracy predictions on different datasets. The F1 score on the training set is 0.873, while the F1 scores on the validation set and test set are 0.861 and 0.868, respectively. This indicates that the model can achieve good classification performance on different datasets. The importance scores of various features affecting IST are demonstrated in Fig. 6. Among them, the ten-fold cross-validation method is used to obtain data with different characteristics, that is, the collected environmental data and tourist behavior data are first integrated into a unified dataset, and the quality and integrity of the data are ensured. Then the whole dataset is divided into ten subsets of equal size to ensure that each subset contains data samples from different time periods or different conditions. In each iteration of cross-validation, one subset is used as the validation set, and the other nine subsets are used as the training set. The data of the training set is used to train the model, and the data of the verification set is used to evaluate the performance of the model, and the performance index of the model on the verification set is recorded. The above steps are repeated ten times, and each time a different verification set is selected, and each subset is ensured to have been used as a verification set and a training set. By calculating the average and standard deviation of each feature, the average performance and uncertainty of performance of each index on different datasets are reflected.

From Fig. 6, temperature is a key characteristic that affects tourist satisfaction, and its characteristics have the highest importance. The increase in temperature is positively correlated with the increase in tourist satisfaction. The amount of snowfall significantly affects the ecological security of IST, and an increase in snowfall may lead to a decrease in ecological security. The number of activities is positively correlated with tourist satisfaction, which may indicate that diversified activities can improve tourist satisfaction. The increase in pedestrian flow may affect the ecological balance of IST, and the importance of features is relatively low. Good air quality is positively correlated with tourist satisfaction, and air quality has a certain impact on tourist experience.

In order to test the effectiveness of the proposed method, classic machine learning algorithms such as Deep Neural Network (DNN), Support Vector Machines (SVM) and Random Forest are selected for comparison. These methods have made remarkable achievements in the fields of environmental monitoring and ecological security prediction. These methods have made remarkable achievements in the fields of environmental monitoring and ecological security prediction, and both of them belong to the category of machine learning algorithm with the algorithm in this paper. Among them, DNN is favored because of its powerful learning ability and efficient representation of complex data, which is suitable for learning large-scale data and complex patterns^48^. SVM is good at dealing with high-dimensional data and nonlinear classification problems, and has good generalization ability and good explanation^49^. As an integrated learning algorithm, random forest can effectively deal with high-dimensional data and a large number of features, and has the ability of anti-over-fitting^50^. Therefore, the comparison of these algorithms can comprehensively evaluate the performance of the proposed method, and it is highly practical and interpretable. Therefore, by introducing these traditional methods into the research, this paper aims to comprehensively evaluate the performance of the proposed comprehensive methods.

**Figure 7:**
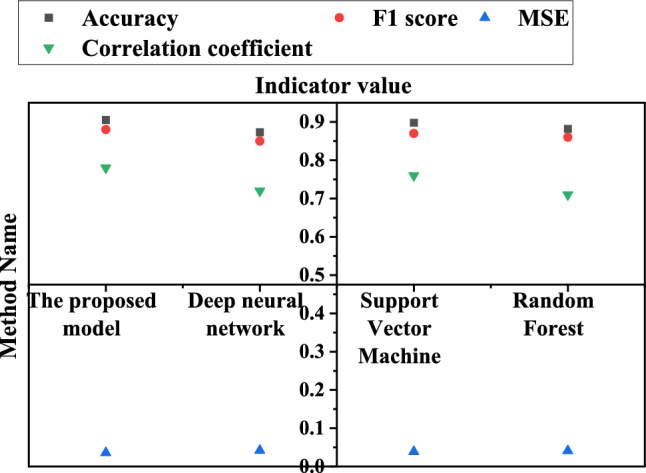
Comparison with different methods.

In Fig. 7, the proposed method exhibits high performance in terms of accuracy, F1 score, and correlation coefficient. The accuracy rate reaches 90.5%, indicating that the model can accurately classify the ecological security and tourist satisfaction of IST. The F1 score is 0.88, considering the accuracy and recall of the model. This suggests that this method performs well in predicting both positive and negative samples. In addition, the MSE is 0.036, indicating that the prediction error of the model is relatively small in regression problems. The correlation coefficient is 0.78, indicating a strong linear relationship between the predicted value of the model and the actual value. This result not only proves the prediction accuracy of the proposed method, but also shows its good fitting degree to the actual situation. What is more noteworthy is that, compared with other comparison methods, the proposed method performs well in these performance indexes. This not only shows the excellent performance of the method compared with the traditional method, but also provides solid data support for the feasibility of the proposed comprehensive research method in practical application. Through these detailed performance indicators, this paper is not only innovative in theory, but also shows obvious advantages in experimental results. This provides strong support for the research and emphasizes the practical application potential of the method in improving the ecological security level of IST and tourists' satisfaction.

## Discussion

Through the comparison of experimental results, the proposed method has shown superiority in multiple performance indicators, such as accuracy, F1 score, and correlation coefficient. This may be because it integrates DL and IoT technology, which can more accurately capture key features in the IST ecosystem and improve prediction accuracy. This is similar to the study by Jena & Behera^51^. The study developed a mathematical model for the tourism supply chain in two scenarios: ecotourism work without and with cost sharing. Zhang et al.^52^ proposed the promotion of artificial intelligence-based tourism demand prediction methods. The study suggested that DL models for predicting tourism demand were often very complex and might encounter overfitting. This was mainly caused by two potential issues: limited access to data and the need for additional explanatory variables. Jain et al.^53^ proposed the use of cuckoo-optimized machine learning models to predict the IST ecological environment. Therefore, this paper can more accurately predict ecosystem changes and fluctuations in tourist satisfaction through real-time monitoring of IoT sensor data, providing scientific decision-making support for tourism managers.

## Conclusion

### Research contribution

The main contribution of this paper is to propose a method that comprehensively utilizes DL models and IoT technology to predict the ecological security and tourist satisfaction of IST. Excellent performance is achieved in the experiment to demonstrate the advantages of the proposed method in multiple performance indicators, such as accuracy, F1 score, MSE, and correlation coefficient. In addition, a detailed feature importance analysis is conducted to reveal the impact of different features on the prediction results to provide more targeted decision-making basis for IST managers.

### Future works and research limitations

The data used here may be affected by collection errors and noise, which may affect the model performance. The experimental data only comes from specific regions and periods and may not be universal. Moreover, the once-in-a-century extreme snowstorm or rainstorm is not considered, which makes the model may not predict accurately when it encounters extreme weather. In addition, all possible influencing factors may not be considered in the feature selection process, and the scope of feature consideration can be further expanded in the future. In the future, more complex DL model architectures and more advanced parameter adjustment methods can be further explored to improve the predictive ability and generalization performance of the model. Subsequent research can also introduce more types of data sources, such as social media and tourist behavior data, to obtain more comprehensive feature information. In the future, more in-depth data analysis will be conducted to reveal possible patterns and trends in the data. The follow-up study should introduce a special handling mechanism for extreme weather conditions into the model, such as introducing a special anomaly detection algorithm or adding additional features to identify and predict ecological security risks under extreme weather conditions. In addition, it is suggested to update the model regularly, constantly monitor and evaluate its performance under different conditions, and retrain the model when new data are available to improve its forecasting ability under extreme weather conditions. Meanwhile, it is planned to adjust and improve the DL model to improve its accuracy and robustness in predicting ecological security and tourist satisfaction.

## Data Availability

The datasets used and/or analysed during the current study available from the corresponding author Baiju Zhang on reasonable request via e-mail zbj@tccu.edu.cn.
